# Inequalities and asymptotics for some moment integrals

**DOI:** 10.1186/s13660-017-1532-7

**Published:** 2017-10-13

**Authors:** Faruk Abi-Khuzam

**Affiliations:** Department of Mathematics, American University of Beirut, Beirut, Lebanon

**Keywords:** 52A20, Ball’s inequality, asymptotics, cube slicing, moments

## Abstract

For $\alpha>\beta-1>0$, we obtain two-sided inequalities for the moment integral $I(\alpha,\beta)=\int_{\mathbb{R}}|x|^{-\beta}|\sin x|^{\alpha}\,dx$. These are then used to give the exact asymptotic behavior of the integral as $\alpha\rightarrow\infty$. The case $I(\alpha,\alpha)$ corresponds to the asymptotics of Ball’s inequality, and $I(\alpha,[\alpha]-1)$ corresponds to a kind of novel “oscillatory” behavior.

## Introduction

Ball’s integral inequality [1], in connection with cube-slicing in $\mathbb{R} ^{n}$, says that for all $s\geq2$,

$$ \int_{-\infty}^{\infty}\biggl\vert \frac{\sin(\pi x)}{\pi x}\biggr\vert ^{s}\,dx \leq \sqrt{ \frac{2}{s}},\quad \text{or}\quad \int_{-\infty}^{\infty}\biggl\vert \frac{\sin x}{x}\biggr\vert ^{s}\,dx \leq\pi\sqrt{\frac{2}{s}}, $$

with strict inequality except when $s=2$. In particular, it suggests that the integral decays like $\frac{1}{\sqrt{s}}$ as $s\rightarrow \infty$, and this is made precise by the following asymptotic [2]:

$$ \lim_{s\rightarrow\infty}\sqrt{\frac{s}{2}} \int_{-\infty}^{ \infty}\biggl\vert \frac{\sin(\pi x)}{\pi x}\biggr\vert ^{s}\,dx= \sqrt{\frac{3}{\pi}}. $$

Since $\sqrt{\frac{3}{\pi}}<1$, the asymptotic result implies the inequality for large values of *s*. But there are no known “easy” proofs of the inequality for the full range of values, the main difficulty being near small values of *s*, e.g., between 2 and 4 [2]. The asymptotic result, though reasonably tame, presents new difficulties when we consider a more general integral, and this is circumvented here by the proof of two new inequalities.

Our purpose here is to consider a generalization involving the “moment” integral

$$ I(\alpha,\beta)= \int_{-\infty}^{\infty}\frac{\vert \sin x\vert ^{\alpha}}{ \vert x\vert ^{\beta}}\,dx,\quad \alpha>\beta-1>0. $$

We shall obtain useful upper and lower bounds for this integral and use them to obtain the asymptotic behavior of this integral. In addition, the inequalities obtained are indispensable in obtaining the asymptotic behavior, especially in the interesting “oscillatory” cases $I(\alpha,[\alpha])$ if $\alpha\geq2$ and $I([\alpha],\alpha-1)$ if $\alpha>1$, where $[\alpha]$ is the greatest integer in *α*. The oscillatory behavior makes it impossible to employ the standard methods used in connection with Ball’s inequality.

We place no restrictions on the indices *α* and *β* beyond those necessary to ensure the convergence of the integral $I(\alpha, \beta)$. Indeed, the condition $\beta>1$ implies convergence in a neighborhood of ∞, and near 0, the inequality $\frac{\vert \sin x\vert ^{\alpha}}{ \vert x\vert ^{\beta}}\leq \vert x\vert ^{\alpha-\beta}$ implies convergence since $\alpha-\beta>-1$.

## Weaker versions of Ball’s inequality

A natural way to deal with Ball’s inequality is to apply the sharp form of the Hausdorff-Young inequality [3]. This leads to two inequalities for the relevant integral: the first works for all $s\geq2$, but falls short of the required inequality by supplying the larger constant $\sqrt{e}$ in place of $\sqrt{2}$. The second gives a constant smaller than $\sqrt{2}$ but only works for $s\geq4$.

### Proposition 1

- *If* $s\geq2$, *then*
  $$ \int_{-\infty}^{\infty} \biggl\vert \frac{\sin(\pi x)}{\pi x} \biggr\vert ^{s}\,dx\leq \frac{1}{ \sqrt{s}}\cdot\sqrt{ \biggl( 1+ \frac{1}{s-1} \biggr) ^{s-1}}< \sqrt{ \frac{e}{s}}. $$
- *If* $s\geq4$, $q=\frac{s}{2}$, *and* *p* *is the index conjugate to* *q*, *then*
  $$ \int_{\mathbb{R} } \biggl\vert \biggl( \frac{\sin\pi\xi}{\pi\xi} \biggr) \biggr\vert ^{s}\,d\xi\leq \sqrt{ \frac{2}{s}} \biggl( \frac{2\sqrt{p}}{p+1} \biggr) ^{q/p}< \sqrt{ \frac{2}{s}}. $$

### Proof

For part (a), let $\chi=\chi_{(-1/2,1/2)}$ be the characteristic function of the interval $(-1/2,1/2)$. Then its Fourier transform is given by $\hat{\chi}(\xi)=\int_{-\infty}^{\infty}\chi(x)e^{-2\pi ix \xi}\,dx=\frac{\sin\pi\xi}{\pi\xi}$. Applying the sharp Hausdorff-Young inequality [3], $\Vert \hat{\chi} \Vert _{s} \leq C_{p}\Vert \chi \Vert _{p}$, where $s\geq2$, $p=s ^{\prime}$, the index conjugate to *s*, and $C_{p}$ is given by $C_{p}^{2}=p^{1/p}(s)^{-1/s}$, we obtain

$$ \int_{R} \biggl\vert \frac{\sin\pi\xi}{\pi\xi} \biggr\vert ^{s}\,d\xi\leq \bigl( p^{1/p}s ^{-1/s} \bigr) ^{s/2}. $$

It now remains to compute

$$ \bigl( p^{1/p}s^{-1/s} \bigr) ^{s/2}=\frac{1}{\sqrt{s}} \cdot \biggl( \frac{s}{s-1} \biggr) ^{\frac{s-1}{2}}=\frac{1}{\sqrt{s}}\cdot \sqrt{ \biggl( 1+\frac{1}{s-1} \biggr) ^{s-1}}< \sqrt{ \frac{e}{s}},\quad s\geq2, $$

and the inequality in (a) follows.

To prove part (b), we employ the convolution $g=\chi\ast \chi$ of the same characteristic function. A simple computation gives

$$ g(x)=\left \{ \begin{matrix} 1-x & 0\leq x\leq1 \\ 1+x & -1\leq x\leq0 \\ 0 & \vert x\vert \geq1 \end{matrix} \right \}. $$

Now $\hat{g}(\xi)= ( \frac{\sin\pi\xi}{\pi\xi} ) ^{2} $, $\Vert g\Vert _{p}^{p}=\frac{2}{p+1}$, and an application of the sharp-Hausdorff-Young inequality gives, for $q \geq2$ and the conjugate index $p=q^{\prime}$,

$$ \begin{aligned} \int_{\mathbb{R} } \biggl\vert \biggl( \frac{\sin\pi\xi}{\pi\xi} \biggr) ^{2} \biggr\vert ^{q}\,d\xi &\leq \bigl( p^{1/p}q^{-1/q} \bigr) ^{q/2} \biggl( \frac{2}{p+1} \biggr) ^{q/p} \\ &=q^{-1/2} \biggl( \frac{2\sqrt{p}}{p+1} \biggr) ^{q/p}< \frac{2 \sqrt{p}}{p+1}\cdot\sqrt{\frac{1}{q}}. \end{aligned} $$

Since $\frac{2\sqrt{p}}{p+1}<1$, we obtain the inequality $\int_{-\infty}^{\infty} \vert \frac{\sin(\pi x)}{\pi x}\vert ^{s}\,dx<\sqrt{ \frac{2}{s}}$ for all $s\geq4$. □

## Main results

In this section we consider the question of obtaining upper and lower bounds for the more general integral, namely $\int_{-\infty}^{\infty }\frac{\vert \sin x\vert ^{\alpha}}{ \vert x\vert ^{\beta}}\, dx$. Those bounds are then used to obtain the precise asymptotic behavior of the integral as $\alpha\rightarrow\infty$. In addition, the bounds make it possible to employ discontinuous functions such as $[\alpha]$ in place of *β*, and then the asymptotic result also captures the precise oscillations in the values of the integral, as $\alpha \rightarrow \infty$.

### Theorem 2

*Suppose* $\alpha>\beta-1>0$, *and put*

$$ I(\alpha,\beta)= \int_{\mathbb{R} }\frac{\vert \sin t\vert ^{\alpha}}{ \vert t\vert ^{\beta}}\,dt=2 \int_{0} ^{\infty}\frac{\vert \sin t\vert ^{\alpha}}{ \vert t\vert ^{\beta}}\,dt, $$

*and*

$$ \phi(\alpha,\beta)=\frac{\alpha^{\frac{\alpha-\beta +1}{2}}\Gamma (\alpha+1)}{\Gamma(\frac{\alpha-\beta+1}{2}+\alpha+1)}, $$

*where* Γ *is the gamma*-*function*. *Then*

$$ \biggl( \frac{6}{\alpha} \biggr) ^{\frac{\alpha-\beta+1}{2}}\Gamma \biggl( \frac{ \alpha-\beta+1}{2} \biggr)\phi(\alpha,\beta)\leq I(\alpha,\beta) \leq \biggl( \frac{6}{\alpha} \biggr) ^{\frac{\alpha-\beta+1}{2}} \Gamma \biggl( \frac{\alpha-\beta+1}{2} \biggr) \biggl\{ 1+\frac{1}{ \beta-1} \biggr\} . $$

In particular, if $\beta=\alpha$, then

$$ \sqrt{\frac{6}{\alpha}}\frac{\sqrt{\alpha}\Gamma(\alpha+1)}{ \Gamma(\alpha+\frac{3}{2})}\sqrt{\pi}\leq I(\alpha,\alpha) \leq \biggl( \sqrt{\frac{6}{\alpha}} \biggr) \sqrt{\pi} \biggl\{ 1+\frac{1}{ \alpha-1} \biggr\} . $$

### Proof

We need first the following double inequality:

$$ 1-\frac{x^{2}}{6}\leq\frac{\sin x}{x}\leq e^{-\frac{x^{2}}{6}},\quad 0 \leq x\leq\pi. $$

The left-hand inequality is easily proved by calculus. It will be used with $0\leq x\leq\sqrt{6}$. For the right-hand inequality, since $0\leq x\leq\pi$, we may use the inequality between the geometric and arithmetic mean of positive numbers to obtain

$$ \prod_{k=1}^{n} \biggl( 1- \frac{x^{2}}{\pi^{2}k^{2}} \biggr) \leq \Biggl( 1-\frac{x^{2}}{\pi^{2}n}\sum _{k=1}^{n}\frac {1}{k^{2}} \Biggr) ^{n} \leq\exp \Biggl( -\frac{x^{2}}{\pi^{2}}\sum_{k=1}^{n} \frac{1}{k ^{2}} \Biggr) . $$

Letting $n\rightarrow\infty$ and recalling the product representation of the sine function and $\sum_{k=1}^{\infty }\frac{1}{k^{2}}=\frac{\pi^{2}}{6}$, we obtain the second inequality. The next step is to compare the full integral in the theorem to an integral over the interval $[0,\sqrt{6}]$, or over $[0,\pi]$.

$$\begin{aligned} \int_{0}^{\sqrt{6}}\frac{\vert \sin t\vert ^{\alpha}}{ \vert t\vert ^{\beta}}\,dt &\leq \int_{0}^{\pi}\frac{\vert \sin t\vert ^{\alpha}}{ \vert t\vert ^{\beta}}\,dt\leq \int_{0} ^{\infty}\frac{\vert \sin t\vert ^{\alpha}}{ \vert t\vert ^{\beta}}\,dt \\ & = \int_{0}^{\pi}\frac{\vert \sin t\vert ^{\alpha}}{ \vert t\vert ^{\beta}}\,dt+\sum _{k=1} ^{\infty} \int_{k\pi}^{(k+1)\pi}\frac{\vert \sin t\vert ^{\alpha}}{ \vert t\vert ^{ \beta}}\,dt \\ & = \int_{0}^{\pi}\frac{\vert \sin t\vert ^{\alpha}}{ \vert t\vert ^{\beta}}\,dt+\sum _{k=1} ^{\infty} \int_{0}^{\pi} \frac{\vert \sin t\vert ^{\alpha}}{ \vert t+k\pi \vert ^{\beta}}\,dt \\ & \leq \Biggl\{ 1+\sum_{k=1}^{\infty} \frac{1}{(k+1)^{\beta}} \Biggr\} \int_{0}^{\pi}\frac{\vert \sin t\vert ^{\alpha}}{ \vert t\vert ^{\beta}}\,dt \\ & \leq \biggl\{ 1+\frac{1}{\beta-1} \biggr\} \int_{0}^{\pi}\frac{ \sin^{\alpha}t}{t^{\beta}}\,dt. \end{aligned}$$

Using the above inequalities for $\frac{\sin x}{x}$,

$$ \int_{0}^{\sqrt{6}}t^{\alpha-\beta} \biggl( 1- \frac{t^{2}}{6} \biggr) ^{\alpha}\,dt\leq \int_{0}^{\pi}\frac{\sin^{\alpha}t}{t^{\beta }}\,dt \leq \int_{0}^{\pi}t^{\alpha-\beta}\exp \biggl( - \frac{\alpha t^{2}}{6} \biggr) \,dt. $$

Simple substitutions to change variables bring this double inequality to the form

$$ \frac{1}{2}6^{\frac{\alpha-\beta+1}{2}} \int_{0}^{1}x^{\frac {\alpha -\beta-1}{2}}(1-x)^{\alpha}\,dx \leq \int_{0}^{\pi}\frac{\sin ^{\alpha }t}{t^{\beta}}\,dt\leq \frac{1}{2} \biggl( \frac{6}{\alpha} \biggr) ^{\frac{ \alpha-\beta+1}{2}} \int_{0}^{\frac{\pi^{2}\alpha}{6}}x^{\frac{ \alpha-\beta-1}{2}}e^{-x}\,dx. $$

If we extend the right most integral to $[0,\infty)$, and then express both sides through the gamma function, we arrive at

$$ \frac{1}{2}6^{\frac{\alpha-\beta+1}{2}}\frac{\Gamma(\frac{\alpha- \beta+1}{2})\Gamma(\alpha+1)}{\Gamma(\frac{\alpha-\beta+1}{2}+ \alpha+1)}\leq \int_{0}^{\pi}\frac{\sin^{\alpha}t}{t^{\beta}}\,dt \leq \frac{1}{2} \biggl( \frac{6}{\alpha} \biggr) ^{\frac{\alpha- \beta+1}{2}}\Gamma \biggl( \frac{\alpha-\beta+1}{2} \biggr). $$

This gives the first inequalities for $I(\alpha,\beta)$, and so, the inequalities for $I(\alpha ,\alpha)$. □

### Corollary 3

*Let* $I(\alpha,\beta)$ *be the integral in the theorem*.

- *If* $\alpha-\beta=c$ *is constant*, *while* $\alpha\rightarrow\infty$, *then*
  $$ \lim_{\alpha\rightarrow\infty}\alpha^{\frac{c+1}{2}}I(\alpha, \beta)=6^{\frac{c+1}{2}} \Gamma \biggl(\frac{c+1}{2} \biggr),\quad c>-1. $$
  *In particular*, *the asymptotic for the integral in Ball’s inequality is*
  $$ \lim_{\alpha\rightarrow\infty}\sqrt{\alpha}I(\alpha,\alpha )=\sqrt{6 \pi}. $$
- *If* $\alpha-\beta=c$, *and* *c* *remains bounded as* $\alpha\rightarrow\infty$, *then*
  $$ I(\alpha,\beta)\backsim \biggl( \frac{6}{\alpha} \biggr) ^{\frac{ \alpha-\beta+1}{2}}\Gamma \biggl( \frac{\alpha-\beta+1}{2} \biggr) , \quad \alpha\rightarrow\infty. $$
  *In particular*,
  $$ I \bigl(\alpha,[\alpha] \bigr)\backsim \biggl( \frac{6}{\alpha} \biggr) ^{\frac{ \alpha-[\alpha]+1}{2}}\Gamma \biggl( \frac{\alpha-[\alpha]+1}{2} \biggr) , \quad \alpha\rightarrow \infty. $$

### Proof

(a) In the very special case where $\beta=\alpha$, Stirling’s formula gives

$$ \phi(\alpha,\alpha)=\frac{\sqrt{\alpha}\Gamma(\alpha+1)}{ \Gamma(\alpha+\frac{3}{2})}\thicksim\frac{\alpha^{\alpha+1}e^{1/2}}{( \alpha+1/2)^{\alpha+1}}= \frac{e^{1/2}}{(1+\frac{1}{2\alpha})^{ \alpha+1}}\thicksim1, \quad \alpha\rightarrow\infty. $$

From this, the case where $\alpha-\beta=c$, a constant, is handled similarly:

$$\begin{aligned} \phi(\alpha,\beta)&=\frac{\alpha^{\frac{c+1}{2}}\Gamma(\alpha+1)}{ \Gamma(\frac{c+1}{2}+\alpha+1)}\thicksim\frac{ \alpha^{\alpha+\frac{c+1}{2}+\frac{1}{2}}e^{-\alpha}}{(\alpha+ \frac{c+1}{2})^{\alpha+\frac{c+1}{2}+\frac{1}{2}}e^{-(\alpha+ \frac{c+1}{2})}} \\ & =\frac{e^{\frac{c+1}{2}}}{(1+\frac{c+1}{2\alpha})^{\alpha+ \frac{c+1}{2}+\frac{1}{2}}}\thicksim1,\quad \alpha\rightarrow\infty. \end{aligned}$$

(c) If $\alpha-\beta=c>-1$, and *c* is only bounded, then Stirling’s formula followed by the inequality $(1+\frac{c+1}{2 \alpha})^{\alpha}\leq e^{\frac{c+1}{2}}$, gives

$$\begin{aligned} \liminf_{\alpha\rightarrow\infty}\phi(\alpha,\beta)&= \liminf_{\alpha\rightarrow\infty} \frac{\alpha^{\frac{c+1}{2}} \Gamma(\alpha+1)}{\Gamma(\frac{c+1}{2}+\alpha+1)} \\ & =\liminf_{\alpha\rightarrow\infty}\frac{e^{\frac {c+1}{2}}}{(1+\frac{c+1}{2 \alpha})^{\alpha+\frac{c+1}{2}+\frac{1}{2}}}\geq \liminf _{\alpha\rightarrow\infty}\frac{1}{(1+\frac{c+1}{2\alpha})^{ \frac{c+1}{2}+\frac{1}{2}}}=1. \end{aligned}$$

So that

$$ \liminf_{\alpha\rightarrow\infty} \biggl[ \biggl( \frac{6}{\alpha} \biggr) ^{\frac{\alpha-\beta+1}{2}}\Gamma \biggl(\frac{ \alpha-\beta+1}{2} \biggr) \biggr] ^{-1}I( \alpha,\beta)\geq1. $$

The corresponding lim sup being clearly ≤1, we obtain

$$ I(\alpha,\beta)\backsim \biggl( \frac{6}{\alpha} \biggr) ^{\frac{ \alpha-\beta+1}{2}}\Gamma \biggl( \frac{\alpha-\beta+1}{2} \biggr) , \quad \alpha-\beta\text{ bounded}, \beta\rightarrow \infty. $$

 □

## Conclusion

For $\alpha>\beta-1>0$, we have obtained two-sided inequalities for the moment integral $I(\alpha,\beta)= \int_{\mathbb{R} }\vert x\vert ^{-\beta} \vert \sin x\vert ^{\alpha}\,dx$ and used them to predict and then prove the exact asymptotic behavior of the integral as $\alpha\rightarrow\infty$. In particular, we showed that the asymptotic behavior $I(\alpha,[\alpha]-1)$ corresponds to the oscillations of $\Gamma ( \frac{\alpha-[\alpha]+2}{2} ) $ between its extreme values. We would like to end by a generalization of the asymptotic result for a class of infinite products. Let *g* be a function having an infinite product representation of the form

$$ g(t)= \prod_{n=1}^{\infty} \biggl( 1- \frac{t^{2}}{t_{n}^{2}} \biggr) , $$

where $t_{n}>0$ and $c=\sum_{n=1}^{\infty}t_{n}^{-2}<\infty$. We are interested in investigating $\lim_{p\rightarrow\infty}\int_{0}^{ \infty}\frac{\vert g(t)\vert ^{p}}{t^{\beta}}\,dt$, where $0\leq\beta<1$. Two examples of such a function are

$$ f(x)= \int_{0}^{1}\cos(xt)h(t)\,dt, \quad\quad J_{0}(x)= \frac{2}{\pi} \int _{0}^{1}\frac{ \cos xt}{\sqrt{1-t^{2}}}\,dt. $$

[Where in the first integral *h* is continuously differentiable, $h(t)>0$, $h^{\prime}(t)<0$, and $h^{\prime\prime}(t)<0$ for $0\leq t\leq1$.] The first function *f* was considered in [4] in connection with maximal measures of sections of the *n*-cube. The second is the Bessel function of order 0.

In studying the asymptotics of $\int_{0}^{\infty}\frac{\vert g(t)\vert ^{p}}{t ^{\beta}}\,dt$, it appears instructive to consider first the case where $\beta=0$.

### Proposition 4

*If* *g* *is the function defined above*, *then we have the double inequality*

$$ 1-ct^{2}\leq \bigl\vert g(t) \bigr\vert \leq e^{-ct^{2}},\quad\quad c= \sum_{k=1}^{\infty}t_{k}^{-2}, $$

*where the left inequality is used when* $0\leq t\leq\frac{1}{ \sqrt{c}}$, *and the right inequality when* $0\leq t\leq t_{1}$. *Furthermore*,

$$ \lim_{p\rightarrow\infty}\sqrt{p} \int_{-\infty}^{\infty } \bigl\vert g(t) \bigr\vert ^{p}\,dt=\sqrt{\frac{ \pi}{c}}. $$

### Proof

In general, if $0< a_{k}<1$, $k=1,2,\ldots,n$, then

$$ 1-(a_{1}+a_{2}+\cdots+a_{n})\leq \prod _{k=1}^{n} ( 1-a_{k} ) \leq \Biggl( 1- \frac{1}{n}\sum_{k=1}^{n}a_{k} \Biggr) ^{n}. $$

The inequality on the left is proved by induction. The inequality on the right is the AGM (arithmetic-geometric mean) inequality. Taking $a_{k}= ( \frac{t}{t_{k}} ) ^{2}$, with $0\leq t\leq t_{1}$, gives

$$ \Biggl( 1-t^{2}\sum_{k=1}^{n}t_{k}^{-2} \Biggr) \leq \prod_{k=1}^{n} \biggl( 1- \frac{t^{2}}{t_{k}^{2}} \biggr) \leq \Biggl( 1-\frac{t^{2}}{n}\sum _{k=1}^{n}t_{k}^{-2} \Biggr) ^{n} \leq\exp \Biggl( -t^{2}\sum _{k=1}^{n}t_{k}^{-2} \Biggr) , $$

and passing to the limit as $n\rightarrow\infty$, we arrive at the required inequalities.

The left-hand inequality gives

$$ \int_{0}^{\infty} \bigl\vert g(t) \bigr\vert ^{p}\,dt\geq \int_{0}^{\frac{1}{\sqrt{c}}} \bigl(1-ct ^{2} \bigr)^{p}\,dt=\frac{1}{2\sqrt{c}} \int_{0}^{1}(1-x)^{p}x^{-1/2}\, dx=\frac{1}{2 \sqrt{c}}\frac{\Gamma(p+1)\sqrt{\pi}}{\Gamma(p+\frac{3}{2})}. $$

By Stirling’s formula we obtain

$$ \liminf_{p\rightarrow\infty}\sqrt{p} \int_{-\infty}^{\infty } \bigl\vert g(t) \bigr\vert ^{p}\,dt \geq\sqrt{\frac{\pi}{c}}, $$

which suggests that the order of decay of the integral is $\frac{1}{ \sqrt{p}}$, and this leads naturally to a consideration of $\sqrt{p}\int_{0}^{\infty} \vert g(t)\vert ^{p}\,dt=\int_{0}^{\infty} \vert g(\frac{t}{ \sqrt{p}})\vert ^{p}\,dt$. Now a substitution in the two-sided inequalities above gives

$$ \biggl(1-c\frac{t^{2}}{p} \biggr)^{p}\leq \biggl\vert g \biggl( \frac{t}{\sqrt{p}} \biggr) \biggr\vert ^{p}\leq e^{-ct ^{2}}, $$

where the left-hand inequality holds true for $0\leq t\leq\frac{ \sqrt{p}}{\sqrt{c}}$, and the right-hand inequality holds true for $0\leq t\leq t_{1}\sqrt{p}$. It now becomes possible to use, exactly as done in [2], Lebesgue’s dominated convergence theorem to conclude that actually $\lim_{p\rightarrow\infty} \sqrt{p} \int_{-\infty}^{\infty} \vert g(t)\vert ^{p}\,dt =\sqrt{\frac{\pi}{c}}$. □

Our final result handles the general case where $0<\beta<1$.

### Theorem 5

*If* *g* *is the function considered above*, *and* $0<\beta<1$, *then*

$$ \lim_{p\rightarrow\infty}p^{\frac{1-\beta}{2}} \int_{0}^{\infty }\frac{\vert g(t)\vert ^{p}}{t ^{\beta}}\,dt= \frac{\Gamma(\frac{1-\beta}{2})}{2 \sqrt{c^{1-\beta}}}. $$

### Proof

In following the same approach as in the proof of Proposition 4 above, we need to know beforehand the expected rate of decay. Thus, using one of the inequalities in Proposition 4, we obtain

$$\begin{aligned} \int_{0}^{\infty}\frac{\vert g(t)\vert ^{p}}{t^{\beta}}\,dt&\geq \int _{0}^{\frac{1}{ \sqrt{c}}} \bigl(1-ct^{2} \bigr)^{p}t^{-\beta}\,dt= \frac{1}{2\sqrt{c^{1-\beta}}} \int_{0}^{1}(1-x)^{p}x^{-\frac {1+\beta }{2}} \,dx \\ & =\frac{1}{2\sqrt{c^{1-\beta}}}\frac{\Gamma(p+1)\Gamma(\frac{1- \beta}{2})}{\Gamma(\frac{1-\beta}{2}+p+1)}, \end{aligned}$$

leading to a sharp lower asymptotic, namely

$$ \liminf_{p\rightarrow\infty}p^{\frac{1-\beta}{2}} \int_{0}^{\infty }\frac{\vert g(t)\vert ^{p}}{t^{\beta}}\,dt\geq \frac{\Gamma(\frac{1-\beta }{2})}{2\sqrt{c ^{1-\beta}}}. $$

Once again this suggests that the expected decay is like $p ^{\frac{\beta-1}{2}}$. So we make the substitution $t=(1- \beta)^{\frac{1}{1-\beta}}p^{-1/2}x^{\frac{1}{1-\beta}}$ and find that

$$ p^{\frac{1-\beta}{2}} \int_{0}^{\infty}\frac{\vert g(t)\vert ^{p}}{t^{\beta }}\,dt= \int_{0}^{\infty} \biggl\vert g \biggl( \frac{(1-\beta)^{\frac{1}{1- \beta}}x^{\frac{1}{1-\beta}}}{\sqrt{p}} \biggr) \biggr\vert ^{p}\,dx. $$

The inequalities

$$ \biggl( 1-c \frac{(1-\beta)^{\frac{2}{1-\beta}}x^{\frac{2}{1-\beta}}}{p} \biggr) ^{p}\leq \biggl\vert g \biggl( \frac{(1-\beta)^{\frac{1}{1-\beta}}x ^{\frac{1}{1-\beta}}}{\sqrt{p}} \biggr) \biggr\vert ^{p} \leq\exp \bigl( -c(1-\beta)^{\frac{2}{1-\beta}}x^{\frac{2}{1- \beta}} \bigr) $$

make it possible to use Lebesgue’s theorem, and we arrive at the asymptotic

$$ \lim_{p\rightarrow\infty}p^{\frac{1-\beta}{2}} \int_{0}^{\infty }\frac{\vert g(t)\vert ^{p}}{t ^{\beta}}\,dt= \frac{\Gamma(\frac{1-\beta}{2})}{2 \sqrt{c^{1-\beta}}}. $$

 □
